# Streamlined SMFA and mosquito dark-feeding regime significantly improve malaria transmission-blocking assay robustness and sensitivity

**DOI:** 10.1186/s12936-019-2663-8

**Published:** 2019-01-25

**Authors:** Tibebu Habtewold, Sofia Tapanelli, Ellen K. G. Masters, Astrid Hoermann, Nikolai Windbichler, George K. Christophides

**Affiliations:** Department of Life Sciences, Imperial College London, London, UK

**Keywords:** *Anopheles coluzzii*, *Anopheles gambiae*, *Plasmodium falciparum*, Malaria, Gametocyte, Standard membrane feeding assay, Mosquito population replacement, Gene drive

## Abstract

**Background:**

The development of malaria transmission-blocking strategies including the generation of malaria refractory mosquitoes to replace the wild populations through means of gene drives hold great promise. The standard membrane feeding assay (SMFA) that involves mosquito feeding on parasitized blood through an artificial membrane system is a vital tool for evaluating the efficacy of transmission-blocking interventions. However, despite the availability of several published protocols, the SMFA remains highly variable and broadly insensitive.

**Methods:**

The SMFA protocol was optimized through coordinated culturing of *Anopheles coluzzii* mosquitoes and *Plasmodium falciparum* parasite coupled with placing mosquitoes under a strict dark regime before, during, and after the gametocyte feed.

**Results:**

A detailed description of essential steps is provided toward synchronized generation of highly fit *An. coluzzii* mosquitoes and *P. falciparum* gametocytes in preparation for an SMFA. A dark-infection regime that emulates the natural vector-parasite interaction system is described, which results in a significant increase in the infection intensity and prevalence. Using this optimal SMFA pipeline, a series of putative transmission-blocking antimicrobial peptides (AMPs) were screened, confirming that melittin and magainin can interfere with *P. falciparum* development in the vector.

**Conclusion:**

A robust SMFA protocol that enhances the evaluation of interventions targeting human malaria transmission in laboratory setting is reported. Melittin and magainin are identified as highly potent antiparasitic AMPs that can be used for the generation of refractory *Anopheles gambiae* mosquitoes.

## Background

A disproportionately high number of global malaria cases (90%) and deaths (91%) are recorded in sub-Saharan Africa, where *Anopheles coluzzii* and *Anopheles gambiae* are the main vectors. The scale-up of vector control interventions in conjunction with early patient diagnosis and therapy has resulted in a substantial reduction in malaria-related cases (21%) and deaths (31%) after 2000. However, the newest data suggest that this progress is progressively coming to a halt or, in fact, being reversed in some countries, indicating that the current malaria intervention tools and strategies may have reached their maximum capacity. This highlights the urgency of developing new complementary technologies targeting malaria transmission in order to achieve the milestones of the new WHO Global Technical Strategy for Malaria 2016–2030 [1].

Research is underway to develop genetically modified mosquito strains that will be able to block or limit the transmission of the deadliest parasite, *Plasmodium falciparum* [2–6]. Such approaches include engineering mosquito strains expressing one or more effector molecules such as AMPs, single chain antibodies against parasite proteins and regulators of the mosquito immune response. The engineered effectors can then be driven through the wild vector population using gene drive technologies such as CRISPR/Cas9 [7–11].

It is vital that such emerging transmission-blocking technologies pass rigorous tests before they are implemented in field trials [12, 13]. An initial critical test is to evaluate in laboratory settings the efficacy of the technologies in reducing mosquito infection by in vitro cultured *P. falciparum*. The ability of an intervention tool to limit malaria transmission in the vector is commonly measured by the reduction in mosquito infection prevalence (proportion of mosquitoes carrying at least one oocyst) and infection intensity (number of oocysts per mosquito) [14]. Previously, it was shown that the two parameters are correlated in both *P. falciparum* and the rodent parasite *Plasmodium berghei* [15].

The standard membrane-feeding assay (SMFA) remains the “gold standard” for evaluating the potential of transmission-blocking technologies including transgenic mosquitoes, antibodies and chemical compounds [16–19]. Due to the difficulty in obtaining consistent and reliable mosquito infections with in vitro cultured *P. falciparum*, SMFA is used by a limited number of laboratories [13, 16, 17]. This difficulty persists despite the existence of numerous published protocols describing the production of *P. falciparum* gametocytes in vitro [20, 21] as well as general mosquito-rearing protocols [22–25]. The complex nature of such experiments involving the mosquito, the parasite, and the microbiota in the mosquito gut, all of which play a critical role in the infection outcome [26, 27], may help to explain this. Therefore, developing a robust SMFA protocol that considers all the aforementioned factors is paramount.

This paper reports a streamlined and robust SMFA protocol that uses *P. falciparum* and *An. coluzzii* to achieve consistently high oocyst intensities and prevalence, thus allowing accurate evaluation of the efficacy of transmission-blocking technologies in a laboratory setting. It provides a thorough description of how gametocyte cultures can be synchronized with mosquito rearing and how mosquito maintenance and feeding regimes can influence the success of infection. Synchronization is important, as *P. falciparum* infectivity is a function of the mosquito age, with the highest infectivity generally observed in 4–5-days old mosquitoes, while older mosquitoes display strong refractoriness to infection. Using this improved SMFA we have tested candidate anti-*Plasmodium* effector peptides designed for genetic engineering of mosquito vectors that can be used in population replacement strategies.

## Methods

### *Anopheles* and *Plasmodium* strains

The *An. coluzzii* N’gousso strain colonized from field-collected mosquitoes of the *An. gambiae* M molecular form in 2006 in Yaoundé, Cameroon, and the *P. falciparum* NF54 strain (MRA-1000, patient E) obtained from the MR4 were used for all the experiments reported in this paper. Mosquitoes were reared and maintained at standard insectary conditions (27 ± 1 °C and 70 ± 5%) humidity on a 12:12 light/dark (L:D) cycle: 11.5 h full light of ~ 300 lx starting at 6 am and 11.5 h darkness starting at 6 p.m., separated by 0.5 h dawn and dusk transitions, respectively. All mosquito infection experiments took place between 2 and 4 p.m.

### Culturing reagents

Complete medium (CM) for *P. falciparum* asexual and gametocyte culture was prepared by mixing 500 mL of sterile liquid RPMI medium (RPMI-1640-R5886, Sigma, UK), hypoxanthine (Sigma-Aldrich, UK) to final concentration of 0.05 g/L, l-glutamine powder (Sigma-Aldrich, UK) to final concentration of 0.3 mg/L and sterile human serum (10% of final volume). CM was aliquoted in 50 mL tubes and stored at 4 °C to be used within a week. Each batch of human serum was tested for supporting the development of healthy gametocytes as determined by optimum exflagellation activity.

Ookinete medium (OM) was prepared by dissolving 500 g of RPMI powder with l-glutamine (Sigma-Aldrich, UK), 2 g NaHCO_3_ and 50 mg of hypoxanthine in 1 L distilled H_2_O (dH_2_O), and stored in sterile 2 L conical flask at 4 °C.

A stock of 100 mM xanthurenic acid (XA) was prepared by adding 205.17 mg XA obtained from Sigma-Aldrich (UK) to 10 mL dH_2_O and 1 mL is added to 1 L ookinete medium. The solution was filtered through 0.2 μm filter units after the pH adjusted to 7.4. It was aliquoted and stored at 4 °C for use within 6 months.

A 10% fructose solution for mosquito sugar feeding was prepared by dissolving 10 g fructose (Sigma, UK) in 1 L distilled water, filtered through a 0.22 μm filter and kept at 4 °C.

### SMFA protocol

**Fig. 1:**
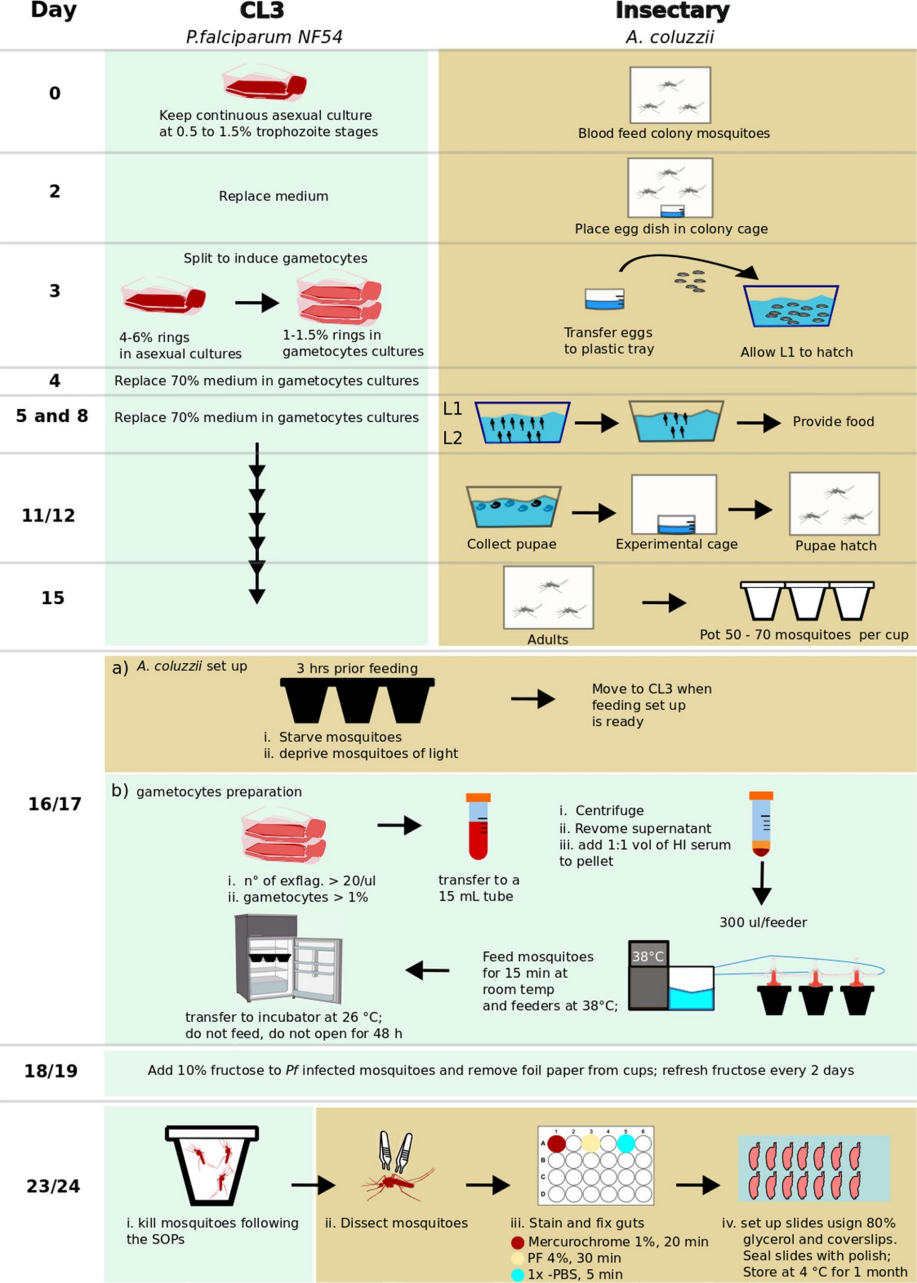
Overview of the protocol for synchronized mosquito and gametocyte culture coupled with the dark-feeding and resting regime

All the steps described below are presented schematically in Fig. 1.

*Day 0. Blood feeding mosquito colony and setting up a Plasmodium falciparum asexual culture* Approximately 50 adult female mosquitoes aged 4–7 days are placed in a clean cage (W24.5 × D24.5 × H24.5 cm, BugDorm, UK) and fed on human blood obtained from the UK National Blood Service. The blood is pre-warmed to a 37 °C and loaded to membrane feeder. The temperature of the blood in the feeder is maintained with reusable hand warmer (Longridge, Amazon, UK). On the same day, *P. falciparum* asexual stage parasites from a source culture running for less than 2 months is diluted to 0.5–1% parasitaemia (trophozoite stage) in 10 mL of CM pre-warmed to 37 °C and 500 μL of A^+^ or O^+^ concentrated red blood cells (cRBCs; NC15 research grade, NHS Blood and Transplant, UK), aliquoted and stored at 4 °C. The cRBCs are derived from a single donor and processed within 15–17 days post drawn.

*Day 1. Parasite medium replacement* Medium replacement is carried out inside Cat 2 microbiological safety cabinet on heat block set at 38 °C. The asexual culture in T25 flask is transferred from the 37 °C incubator to heat block without agitating the cells. The medium is aspirated and replaced with 10 mL fresh CM, pre-warmed to 37 °C. The culture is returned to the incubator after fumigation for 15 s with a gas mixture consisting 5% O_2_, 5% CO_2_ and 90% N_2_.

*Day 2. Oviposition dish setup and parasite medium replacement* A flat bottom 200 mL glass dish half-filled with 0.01% NaCl, where the tip of a funnel-shaped folded 45 mm Whatman filter paper is dipped, is placed in the mosquito cage which was blood fed 28 h earlier. On the same day, the medium in the *Plasmodium* asexual culture is replaced as described above.

*Day 3. Egg floating and gametocyte induction* The oviposition dish is removed from the cage and the eggs are washed off the filter paper into a beaker containing 1% NaOCl. After 5 min, the eggs are rinsed twice in dH_2_O and floated in 1.5 L salt water (0.01%) in a transparent plastic tray (W45 × D45 × H30 cm) lined with Whatman paper. The salt water is dusted with pulverized fish food (Nishkoi Health, Swell, UK) and the tray is covered with a mosquito net.

A gametocyte culture is initiated from an asexual stage culture of 4–6% early ring stages. First, a subsample (300–500 μL) of the asexual parasite culture is collected from the flask and pelleted by quick spinning. The pellet is used to prepare a thin smear on glass slide, stained using the Kwik-Diff™ Stains kit (Thermo Scientific, UK) and examined for synchrony. When the population of young rings reaches an optimum, the culture is diluted in prewarmed (37 °C) 10 mL of CM supplemented with 500 μL cRBC in a T25 flask to a final concentration of young merozoites of 1–1.5%. Upon fumigation with the O_2_/CO_2_/N_2_ gas mixture for 15 s, the culture flask is immediately placed in a 37 °C incubator.

*Day 4. Medium change of gametocyte culture* The gametocyte culture is maintained by daily replacement of 70% of the CM (from Day 4 onward). During CM replacement, the old medium is carefully aspirated without disturbing the infected RBC layer at the bottom of the flask and fresh CM is gently added as described above. The flask is fumigated with gas mixture for 15 s and returned to the incubator.

*Days 5*–*7. Thinning the first instar (L1) larvae culture* To initiate larval hatching the tray with floated eggs is sprinkled with cold dH_2_O. After about 1 h, the newly hatched larvae (L1) are split to a density of ~ 1600 larvae per tray containing 1.5 L dH_2_O. The larval water is dusted with 0.6 g pulverized food pellet and the tray is covered with net. From day 5 to 7, the larvae are fed double the amount of the previous day.

*Days 8. Second larvae thinning* Early stage III larvae (L3) are spilt to a density of ~ 200 L3 per tray with dH_2_O. Trays are covered with net after addition of 1.8 g food pellet. Food pellets are added every day as long as the water is clear and contains no un-dissolved pellets.

*Days 11*–*12. Pupae collection* The contents of each tray are transferred into a 2 L bottom round bulb flask till just below the brim. After 3 min, pupae concentrated on the top are removed by tipping in a strainer. Larvae that remain at the bottom of the flask are returned to a clean tray, provided with fresh dH_2_O and food pellets and are left for another round of pupation for collection on the next day. While still in the strainer, pupae are rinsed with dH_2_O and transferred to a clean 200 mL glass beaker that is then half filled with dH_2_O and placed in an adult cage containing cotton wool soaked in 10% fructose solution. The pupae dish is removed the next day and/or once adult mosquitoes eclose.

*Day 15. Mosquito potting* mouth operated aspirator is used to transfer 50–60 female mosquitoes to 500 mL volume paper cup covered with a double layer of netting via a hole cut at the side, and the cup side opening is sealed with tape. A cotton ball soaked in 10% fructose is placed on top of the net. Mosquito cups are stored in a secondary container box in the insectary.

*Day 16/17. Gametocyte feeding* A 14/15-day old gametocyte culture is used for mosquito infections. Three hours before gametocyte feed, the fructose-soaked cotton ball is removed from the cups to starve the mosquitoes, and mosquito cups are placed in the dark inside a second container for biosafety reasons. The container is placed in an incubator set at standard insectary conditions described above.

One hour before feeding heat-inactivated human serum (56 °C for 30 min) is defrosted at RT and placed on heat block at 37 °C. An aliquot of cRBCs (200 μL) is transferred to a 15 mL sterile tube and placed on the heat block. A water bath in the mosquito gametocyte feeding station is switched on and set at 38 °C to pre-warm membrane feeders. A membrane feeder is set up as follows: an approximately 5 cm × 5 cm parafilm (Brand®Parafilm® M, sealing film) piece is stretched carefully in all directions and pressed onto the outer rim of the glass feeder chamber in order to seal the chamber. The excess film is attached onto the water jacket of the feeder.

The gametocyte culture is hand-swirled and placed on 37 °C heat block. A sample of 500 μL is aspirated using pre-warmed pipette, transferred to a 1.5 mL tube and centrifuged at 2000*g* for 30 s at RT (21 °C). A thin smear is prepared on the glass-slide from a drop of the culture precipitate, air-dried and stained using the Kwik-Diff™ Stains kit (Thermo Scientific, UK) to evaluate the count mature (stage V) gametocytes. The remaining precipitate is resuspended in 30 μL OM, and a 10 μL suspension is loaded into a FastRead™ chamber and incubated for ~ 20 min at RT. The number of exflagellation centres formed in the 4 × 4 grid is determined under the microscope (10× objective).

After removing 50% of the CM, the remaining culture containing 500 μL of gametocytaemic blood (gametocytes concentration may vary from 1 to 6% per T25 flask) is transferred to the pre-warmed 15 mL tube containing the cRBCs (200 μL of cRBCs in every 1 mL of gametocytaemic blood culture). In 10 randomly selected mosquito infection experiments, the final average gametocytaemia in the *P. falciparum* infected blood meal was 2.1% (SD, 0.5). The tube is spun at 6000*g* for 5 min at 37 °C and the resulting pellet is resuspended in pre-warmed heat-inactivated serum (1:1 v/v). An aliquot of 300–500 μL mixture is loaded in a pre-warmed membrane feeder. Mosquitoes are allowed to feed on the membrane feeder for 15–20 min at room temperature (21 °C) in the dark. After completion of the feed, the cups are placed in a dark secondary box container that is placed in an incubator set at 27 °C with 60–70% RH and standard 12:12 L:D cycle. Next morning, i.e. 12–18 h after the feed, the cups are transferred to a transparent secondary box to allow mosquitoes be exposed to the standard L:D regime. Cotton pads soaked in 10% fructose are placed on the cups only 48 h after the feed so that non-blood fed mosquitoes are starved to death. Cotton pads are replaced every 2 days until the day of dissection.

*Day 23/24. Dissection and examination of mosquito midguts* Seven days post-infection, mosquitoes are dissected in PBS supplemented with 1% NaN_3_. The dissected guts are stained with 0.5% mercurochrome for 10 min, fixed in 4% paraformaldehyde for 30 min and washed in PBS for 30 min twice. Guts are mounted on glass slides in 80% glycerol and examined under a light microscope (20×) for oocyst presence.

### Evaluation of the efficacy of anti-*Plasmodium* AMPs

AMPs were synthesized by GenScript USA Inc. Before mosquito infection, AMPs were dissolved in distilled PBS and warmed up at 37 °C on a heat block. Stock AMP solution was added to 300–500 μL of gametocytaemic blood mix prepared as described above at the desirable concentration and mixed by pipetting. Calculation of compound concentrations was performed accordingly to the volume of blood mix used. An equivalent volume of PBS was mixed to the control blood mix. The blood mixtures were loaded into a feeder using pre-warmed 1 mL tips.

### Data analysis

Data on the wing length were analysed using Student’s t test in GraphPad Prism7 software. Oocyst counts were analysed by Wald Z-test on a zero-inflated negative binomial regression using generalised linear mixed model (GLMM) in R (version 2.15.3). Oocyst prevalence data were analysed by χ^2^ test in the GraphPad Prism7.

## Results

### Dark-feeding increases mosquito infection and SMFA sensitivity

**Fig. 2:**
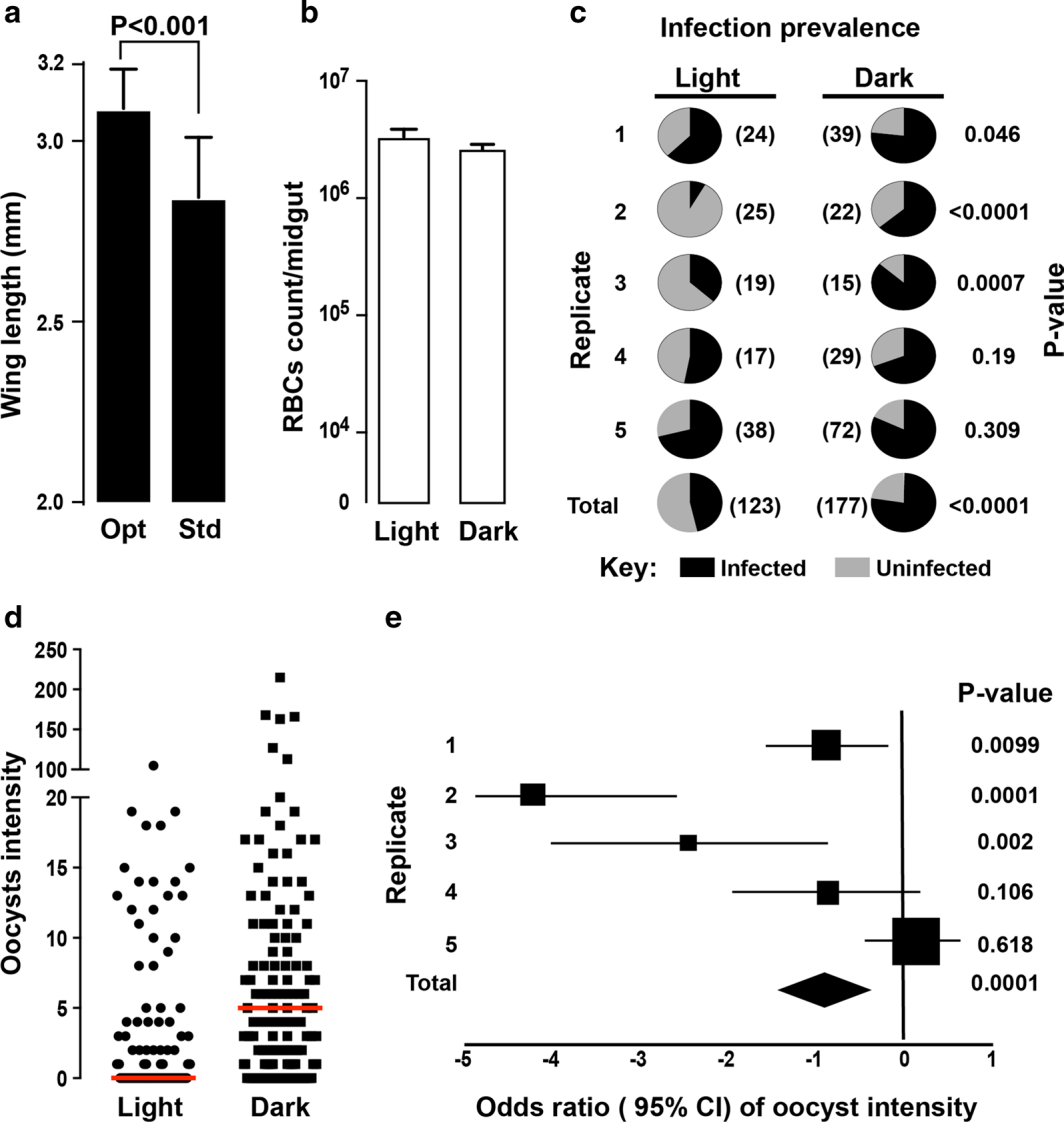
Mosquito infection with *Plasmodium falciparum* using the streamlined SMFA and mosquito dark-feeding and resting regime. **a** Comparison of wing length as a proxy for body size between mosquitoes reared as reported in this manuscript (optimal; opt) and mosquitoes generated under standard colony maintaining regime (std). **b** Comparison of RBC count in the mosquito gut bolus, used as a proxy to bloodmeal volume, between mosquitoes maintained in dark conditions for 3 h prior to, during and several hours after blood feeding (dark-feeding regime) compared mosquitoes fed using the standard SMFA protocol. **c** Pie-charts showing *P. falciparum* oocyst infection prevalence in mosquitoes under dark-feeding regime compared to mosquitoes fed using the standard SMFA protocol. Numbers in brackets show the number of mosquitoes used for each replicate as well as the total number of mosquitoes. **d** Overall *P. falciparum* infection intensities between mosquitoes under dark-feeding regime and mosquitoes fed using the standard SMFA protocol. **e** Forest plot showing estimate of odds ratio (± 95% CI) of oocyst intensities between mosquitoes under a dark-feeding regime and control. Squares and diamond shows the sample size for each replicate and the total, respectively

The optimized mosquito husbandry described in the Methods section and schematically presented in Fig. 1 resulted in mosquitoes with significantly larger body size as measured by the wing length compared to mosquitoes generated with standard colony rearing (Fig. 2a; P < 0.001). Comparison of the coefficients of variations (*CV*) of the wing length revealed that the test mosquitoes (*CV *= 3.49%) were 1.7 times more uniform compared to the standard colony mosquitoes (*CV *= 5.85%).

The impact of dark and light feeding regimes on *P. falciparum* infectivity to *A. coluzzii* mosquitoes was evaluated. Specifically, on the day of feeding, mosquito cups were placed in the dark 3 h prior to the feed (test cups) or kept at standard insectary light regime (control cups). During the feed, test cups were fully covered with aluminum foil whereas control cups were not. Immediately after the completion of blood feeding, subsamples of 15 blood-fed mosquitoes were removed from the test and control cups, respectively, and the volume of their bloodmeal uptake was measured. No difference in RBC counts per midgut was detected between test and control mosquitoes (Fig. 2b).

The test cups were placed in a dark secondary container box and control cups were placed in a transparent box. Both boxes were placed in an incubator set at 26 °C, 60–70% RH and standard L:D cycle. Eighteen (18) hours post feeding, the test cups were removed from the dark box and placed in the transparent box together with the control cups. Five independent replicate feeds were performed, and the results are presented in Fig. 2c–e. Analysis of the results showed that the dark-feeding regime significantly increases both the oocyst prevalence (P < 0.001) and infection intensity (P < 0.001) compared to feeding under standard light conditions.

### New SMFA pipeline provides increased performance and reliability

**Fig. 3:**
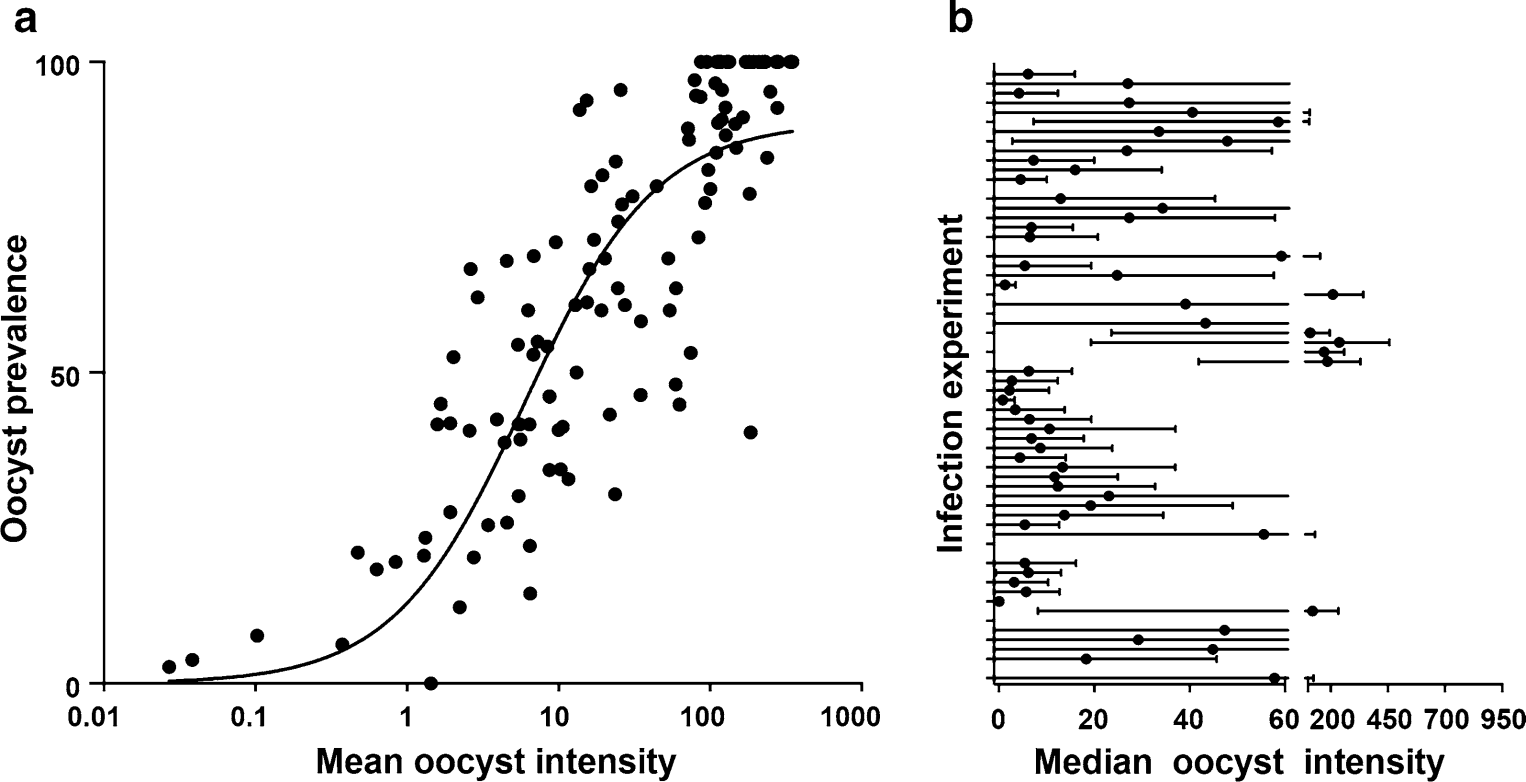
Summary of infections obtained using synchronized mosquito and *Plasmodium falciparum* gametocyte culture and the dark-feeding regime. **a** Scatter plot of mean infection intensities versus prevalence (%) for 123 feeds that used a total of 2703 mosquitoes. The fitting line was generate using Sigmoidal 4PL analysis, X is log (oocyst count) and Y is the prevalence: Y = 86.65/(1 + 10^(− 2.73 − 0.03X)^). **b** Parasite infection intensity for 63 control feeds (no further treatment). Dots and lines represent median and range, respectively

The coordinated mosquito and gametocyte culturing approach together with the mosquito dark-feeding regime were applied to 124 infection experiments over the course of 18 months. The data generated from these infections showed a strong, positive yet nonlinear relationship between the infection intensity and prevalence (Fig. 3a), in agreement with previous works [28, 29]. Analysis of 64 of these infections in which neither the mosquitoes nor the gametocytes were treated (control feeds) is presented in Fig. 3b. The results showed an average infection intensity and prevalence of 40.1 (SEM ± 6.4) and 52% (SEM ± 6.2), respectively. These measures meet the previously recommended standards for reliably performing transmission-blocking assays [15, 30].

### Screening of AMP peptides reveals strong effects of Magainin and Melittin

**Fig. 4:**
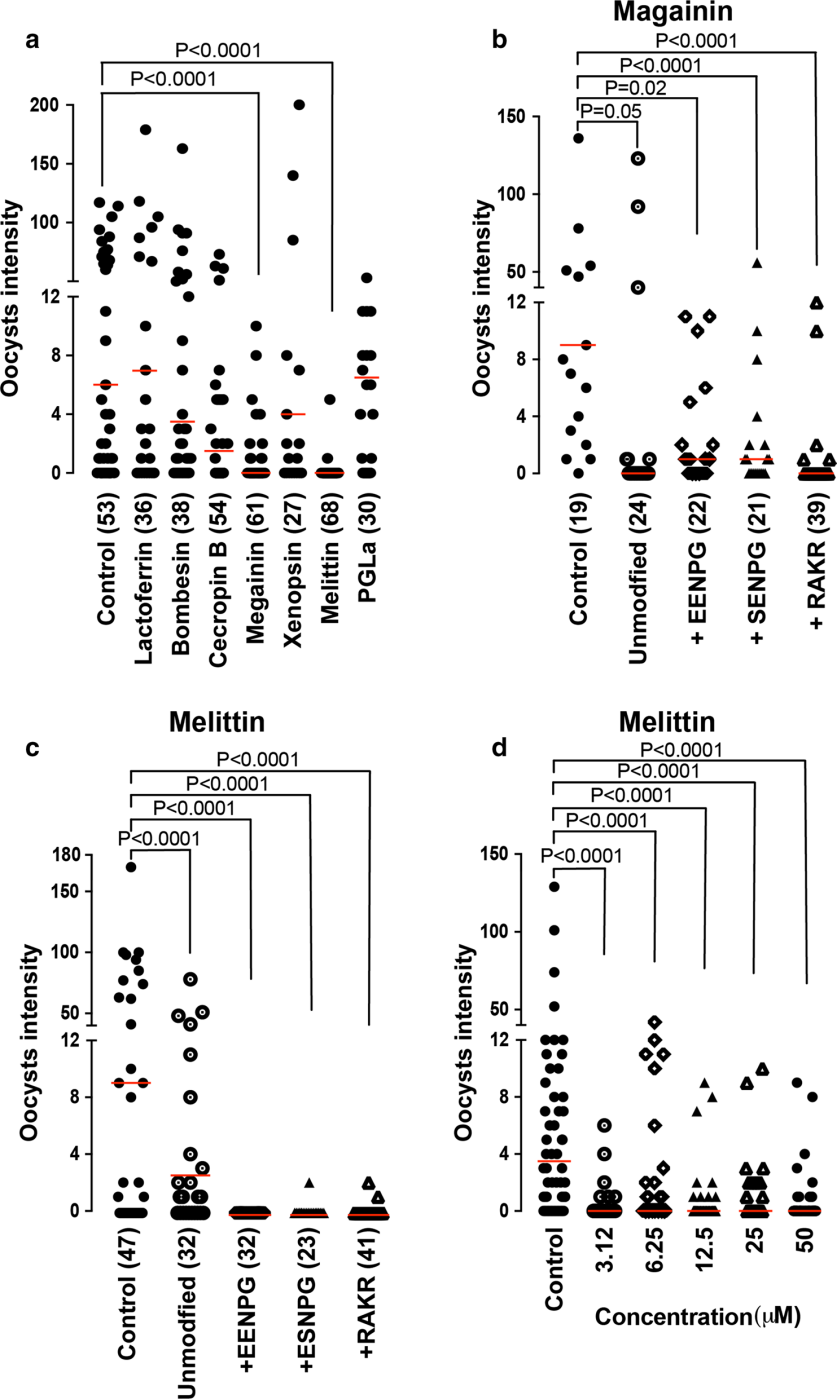
Effect of AMPs on *Anopheles coluzzii* infections with *Plasmodium falciparum*. **a** Infection intensity in mosquitoes fed on cultured *P. falciparum* gametocytes spiked with water (control), and AMPs at a 50 μM dose. **b**, **c** Infection intensity in mosquitoes fed on cultured gametocytes spiked with modified magainin and melittin, respectively. **d** Infection intensity in mosquitoes fed on cultured gametocytes spiked with different concentration of melittin-EENPG. Dots shows oocyst counts for individual midguts and red lines show median infection intensities

The developed SMFA pipeline was used to screen the transmission blocking potential of six selected AMPs, including Bombesin of the European fire-bellied toad *Bombina bombina*, Cecropin B of the cecropia moth *Hyalophora cecropia*, Melittin of the western honeybee *Apis mellifera*, and magainin, xenopsin and PGLa of the African clawed frog *Xenopus laevis*. These molecules have previously been reported to exhibit varying levels of microbicidal properties against blood stage *P. falciparum* [31–34]. The six AMPs were added to the gametocytaemic blood mix that was fed to mosquitoes at 50 μM final concentrations. We observed a significant transmission-blocking activity by melittin (P < 0.001) and magainin (P < 0.001) compared to control, PBS-treated mosquitoes (Fig. 4a). These results suggested that magainin and melittin could be developed as effector genes for expression in transgenic mosquito strains.

It was next tested whether the two AMPs would remain active when expressed in transgenic mosquitoes in the context of multi-AMP arrays separated via 2A auto-cleavage peptides or a furin cleavage site. Processed peptides following the 2A or furin cleavage are expected to carry additional amino acids that can alter the peptide net charge. Specifically, the amino acid sequences EENPG and ESNPG would remain at the C-terminus of the upstream peptide in the case of T2A or P2A and E2A or F2A processing signals, respectively, while a single proline would remain at the N-terminus of the downstream peptide in both cases [35, 36]. Similarly, following introduction and cleavage of a furin site, the C-terminal RAKR and N-terminal AP amino acid sequences would be added to the upstream and downstream peptides, respectively.

The effect of the C-terminal EENPG, ESNPG and RAKR modifications on the ability of the two peptides to inhibit parasite development in the mosquito was tested. The results showed that the modifications have no significant negative effect on the transmission-blocking activity of magainin (Fig. 4b) or melittin (Fig. 4c). What is more, the C-terminal EENPG modification appeared to enhance the anti-parasitic activity of melittin at concentrations as low as 3.1 μM (Fig. 4d).

## Discussion

The SMFA is subject to both inter-feed and intra-feed variability, posing a difficulty for interpreting data generated to test malaria transmission-blocking interventions. This underlines an urgent need for an improved and optimized SMFA protocol to increase the assay sensitivity and reliability [30]. Several SMFA protocols have been developed since the publication of the first general laboratory infection protocol [37, 38]. Such protocols are generally focused on the *P. falciparum* gametocyte culturing system in a temperature-controlled environment with regimented procedures specifically describing the techniques to obtain viable gametocytes [20, 21]. Obtaining mature gametocytes (stage V) with an optimized and efficient method is critical for a successful SFMA [39]. Nonetheless, such practices alone cannot guarantee successful mosquito infection as infection variability remains.

To date, SMFA protocols do not sufficiently consider mosquito factors affecting parasite infectivity, including mosquito physiology and fitness [40, 41], which are in turn influenced by factors such as the composition and load of microbiota in the gut and the mosquito immune response [42–44]. For example, it has been shown that larval diet can greatly affect mosquito fitness as well as the load and composition of gut enterobacteria and their permissiveness to *Plasmodium* [41, 45, 46]. Mosquitoes with larger body size, often considered as an indicator for fitness, have an increased *Plasmodium* infection intensity and prevalence, which is attributed to better post-infection survival. Additionally, obtaining fit experimental mosquitoes ensures that enough mosquitoes survive until dissection.

The comprehensive SMFA protocol for experimental infections of *An. coluzzii* mosquitoes with cultured *P. falciparum* parasites reported here is based on careful mosquito husbandry and streamlined parasite culturing, synchronized culturing of the two organisms and mosquito feeding under dark conditions. Mosquito husbandry was designed to mitigate factors that negatively affect fitness by controlling the availability of larval food and population density. Implementing this regiment consistently allowed generating homogeneously fit mosquitoes for the infection experiments. Parasite culturing is focused on removing unnecessary steps from previous protocols, such as heat inactivating human serum for medium preparation, pooling of day 16 and day 18 gametocyte cultures, and removal of unfed mosquitoes after blood feed which requires keeping the mosquitoes on ice (chilling) for a significant time period [15, 17]. These can minimize human and other technical errors in carrying out this labor-intensive protocol. The synchronization of mosquito and parasite culturing ensures that appropriately aged mosquitoes are available at the time gametocytes are ready for infection. Finally, and perhaps most importantly, the dark-feeding regime is designed to emulate the natural conditions upon which *An. coluzzii* feed on gametocyte carriers, considering also the fact that these mostly indoor-resting mosquitoes remain inside houses, in close to dark conditions for prolonged time following blood feeding. It is shown that the dark-feeding and resting regime can consistently provide high infection outcomes with regards to both infection intensity and prevalence, both of which are key parameters for SMFA performance [28]. The improved SMFA protocol reported here has led to 68.5% (85/124) of all infections showing infection intensity of ≥ 5 and prevalence of ≥ 50%, a benchmark previously reported to allow accurate evaluation of the efficacy of transmission-blocking interventions [15, 28].

Using the new SMFA pipeline, the transmission-blocking activity of six cationic AMPs previously reported to exhibit plasmodicidal properties was evaluated, with the aim to select the most active peptides for engineered expression as anti-parasitic effectors in the gut of transgenic mosquitoes designed to reduce malaria transmission. Such effector can be driven through wild mosquito populations through CRISPR/Cas9-mediated gene drive. The dominant view regarding the mechanism of action of cationic AMPs is that they initially interact with the negatively charged lipid-bilayer of the cell membrane and then assume an amphipathic helical conformation that induces insertion of the hydrophobic motif into the bilayer causing loss of membrane integrity and, ultimately, cell lysis [49, 50]. AMPs are preferentially active against prokaryotic cells due to their higher negative surface potential compared to eukaryotes [51]. Most AMPs can retain their activities in heterologous host systems, which makes them suitable candidates for biotechnological engineering of pathogen resistance in transgenic plants and animals. Studies have demonstrated that high expression of transgene AMPs can be attained without any negative effect on the host organism [47]. Due to these characteristics, the transgenic expression of AMPs in plants and animals is emerging as one of the most promising platforms for disease control.

AMPs from various sources with different levels of plasmodicidal activities have been previously discovered [33, 48–52]. Using this assay, it is demonstrated that two of these AMPs, melittin from the western honeybee *Apis mellifera* and magainin from the African clawed frog *Xenopus laevis*, exhibit very strong anti-parasitic properties against *P. falciparum* when present in the gut of *An. coluzzii*. Both AMPs were previously shown to induce parasite killing. Injection of magainin into anopheline mosquitoes previously infected with different *Plasmodium* species was shown to completely block parasite sporogony [49]. Melittin was shown to kill in vitro produced *P. berghei* ookinetes at a 50 μM concentration and to also lead to a significant decrease in infection prevalence and intensity in both *An. coluzzii* and *Anopheles stephensi* [34]. When mixed with gametocytic blood, melittin was shown to reduce *P. falciparum* oocyst prevalence and intensity in *An. coluzzii*.

Among such mosquito genetic modification strategies is expression of polycistronic mRNAs encoding a series of AMPs in the gut, which would be separated through either self-cleaving 2A peptides or enzymatic cleavage. In this direction, it is shown that the amino acid tags left by those processes at the C-terminus of both melittin and magainin do not affect their anti-parasitic activities and indeed could further enhance them, as is the case of melittin-EENPG that is produced by T2A and P2A self-cleavage. These data provide significant insights into ongoing efforts to engineer mosquitoes that would be able to block human malaria transmission.

## Conclusion

This paper reports a streamlined and robust SMFA protocol for laboratory infection of *An. coluzzii* with cultured *P. falciparum* gametocyte. It provides a thorough description of how gametocyte cultures can be synchronized with mosquito rearing and how mosquito maintenance and feeding regimes can influence the success of infection. Importantly, it demonstrates that infecting and keeping mosquitoes in dark conditions can significantly enhance the infection outcome. This revised SMFA protocol consistently produces high oocyst intensities and prevalence, allowing a sensitive and accurate evaluation of the efficacy of transmission-blocking technologies in a laboratory setting. Using this protocol, it is demonstrated that melittin and magainin exhibit strong transmission blocking properties against *P. falciparum* when present in the *An. coluzzii* midgut and are good candidates for expression in transgenic mosquitoes that can be used in mosquito population replacement strategies.

## Abbreviations

SMFA: standard membrane feeding assay
AMPs: antimicrobial peptides
SEM: standard error of the mean
